# Supplementary material for: Reconciling Mining with the Conservation of Cave Biodiversity: A Quantitative Baseline to Help Establish Conservation Priorities
Source: PLoS One. 2016 Dec 20;11(12):e0168348. doi: 10.1371/journal.pone.0168348 (PMC5173368; doi:10.1371/journal.pone.0168348)
Supplement: S1 Dataset — (ZIP) [file pone.0168348.s002.zip › Taxa/Serra Norte/SN_2013/N5S/Lista N5S-02.pdf]

CAVIDADE N5S-0002

Classe	Ordem	Fam/Outros	Gên/Outros	Espécie	S-U	U-S	Zon.
Arachnida	Acari	Ixodidae		jovem	X		E
Arachnida	Amblypygi	Phrynidae	*Heterophrynus*	*longicornis*		X	
Arachnida	Araneae	Araneidae	*Alpaida*	*antonio*	X		P
Arachnida	Araneae	Araneidae	*Mangora*	sp.2	1		E
Arachnida	Araneae	Araneidae	*Parawixia*	*kochi*	X		E
Arachnida	Araneae	Ctenidae	*Ctenus*	sp.2	1		P
Arachnida	Araneae	Drymusidae	*Drymusa*	*spelunca*	X	X	E
Arachnida	Araneae	Filistatidae		jovem	X		E
Arachnida	Araneae	Gnaphosidae		jovem		X	
Arachnida	Araneae	Oonopidae		jovem	X		E
Arachnida	Araneae	Pholcidae	*Mesabolivar*	*aurantiacus*	X		E
Arachnida	Araneae	Pholcidae	*Mesabolivar*	sp.1	X		E/P
Arachnida	Araneae	Pholcidae		jovem	X	X	E
Arachnida	Araneae	Salticidae		sp.9	X		E
Arachnida	Araneae	Scytodidae	*Scytodes*	sp.		X	
Arachnida	Araneae	Segestriidae	*Ariadna*	sp.		X	
Arachnida	Araneae	Tetragnathidae	*Azilia*	*histrio*	X		E/P
Arachnida	Araneae	Theridiidae	*Nesticodes*	*rufipes*		X	
Arachnida	Araneae	Theridiidae	*Theridion*	sp.1	X	X	E
Arachnida	Araneae	Theridiosomatidae	*Plato*	sp.1	X	X	P
Arachnida	Araneae	Uloboridae		jovem	X		E
Arachnida	Pseudoscorpiones	Chernetidae		jovem	X	X	P
Arachnida	Pseudoscorpiones	Chthoniidae	*Pseudochthonius*	sp.1	X		E
Chilopoda	Scolopendromorpha	Cryptopidae	*Cryptops*	jovem	1		E
Chilopoda	Scolopendromorpha	Scolopocryptopidae	*Newportia*	sp.1	1		E
Chilopoda	Scutigeromorpha	Pselliodidae	*Sphendononema*	*guildingii*	1	X	E
Entognatha	Collembola	Paronellidae		sp.1	X	X	P
Insecta	Blattodea			jovem	1	X	E
Insecta	Coleoptera	Scydmaenidae		sp.		X	
Insecta	Coleoptera	Staphylinidae	Scaphidiinae	sp.1	X		E
Insecta	Coleoptera	Tenebrionidae	Alleculinae	jovem		X	
Insecta	Diptera	Cecidomyiidae	Cecidomyiinae	sp.	X		E
Insecta	Diptera	Chironomidae		sp.	X		E
Insecta	Diptera	Culicidae	*Culex*	sp.	X	X	E
Insecta	Diptera	Nematocera		sp.		X	
Insecta	Diptera	Psychodidae	Phlebotominae	sp.	X		E/P
Insecta	Diptera	Sciaridae		sp.	X		E
Insecta	Diptera	Tipulidae	Tipulinae	sp.	X		E
Insecta	Heteroptera	Lygaeidae		jovem	X		E
Insecta	Heteroptera	Reduviidae		jovem		X	
Insecta	Homoptera	Cixiidae		jovem		X	
Insecta	Hymenoptera	Chalcidoidea		sp.		X	
Insecta	Hymenoptera	Formicidae	*Camponotus*	sp.2	X	X	P
Insecta	Hymenoptera	Formicidae	*Crematogaster*	sp.1	X	X	E
Insecta	Hymenoptera	Formicidae	*Dolichoderus*	aff. *bispinosus*	X		E
Insecta	Hymenoptera	Formicidae	*Gnamptogenys*	sp.1	X	X	E
Insecta	Hymenoptera	Formicidae	*Paratrechina*	sp.		X	
Insecta	Hymenoptera	Formicidae	*Pheidole*	sp.		X	
Insecta	Isoptera	Termitidae	*Nasutitermes*	sp.	X		E

Insecta	Lepidoptera	Gelechioidea		sp.	X		E
Insecta	Lepidoptera	Noctuoidea		sp.3	1		E
Insecta	Orthoptera	Phalangopsidae	*Paraclodes*	sp.	6	X	E
Insecta	Orthoptera	Phalangopsidae	*Phalangopsis*	sp.	6	X	
Insecta	Psocoptera	Epipsocidae	*Epipsocus*	sp.2	X		P
Insecta	Psocoptera	Manicapsocidae	*Nothoentomum*	sp.1	X		E
Insecta	Psocoptera	Psyllipsocidae	*Psyllipsocus*	sp.1	X		E
Insecta	Psocoptera	Ptiloneuridae	*Triplocania*	sp.9	X		E
Insecta	Psocoptera			jovem		X	
Gastropoda	Pulmonata	Systrophiidae	*Happia*	sp.		X	
Malacostraca	Isopoda	Philosciidae		sp.		X	
Symphyla		Scutigerellidae		sp.		X	
Amphibia	Anura	Strabomantidae	*Pristimantis*	cf. *fenestratus*		1	P
Mammalia	Chiroptera	Emballonuridae	*Peropteryx*	*kappleri*	4		E
Reptilia	Squamata	Gekkonidae	*Thecadactylus*	sp.	2		E
Reptilia	Squamata	Serpentes		sp.	1		E
